# Investigation of Prenatal Pesticide Exposure and Neurodevelopmental Deficits in Northern Thailand: Protocol for a Longitudinal Birth Cohort Study

**DOI:** 10.2196/31696

**Published:** 2022-02-07

**Authors:** Brittney O Baumert, Nancy Fiedler, Tippawan Prapamontol, Panrapee Suttiwan, Warangkana Naksen, Parinya Panuwet, Supattra Sittiwang, Chayada Dokjunyam, Melissa M Smarr, Carmen J Marsit, P Barry Ryan, Wattasit Siriwong, Mark G Robson, Dana Boyd Barr

**Affiliations:** 1 Gangarosa Department of Environmental Health Rollins School of Public Health Emory University Atlanta, GA United States; 2 Environmental and Occupational Health Sciences Institute Rutgers University Piscataway, NJ United States; 3 Research Institute for Health Sciences Chiang Mai University Chiang Mai Thailand; 4 Faculty of Psychology Chulalongkorn University Bangkok Thailand; 5 Faculty of Public Health Chiang Mai University Chiang Mai Thailand; 6 College of Public Health Sciences Chulalongkorn University Bangkok Thailand; 7 School of Environmental and Biological Sciences Rutgers University Piscataway, NJ United States

**Keywords:** birth cohort, farmworker, neurodevelopment, organophosphate, pesticide, pregnant women, Thailand

## Abstract

**Background:**

Prenatal exposure to pesticides has been linked to adverse neurodevelopmental outcomes. Gaps exist in the current literature about the timing and magnitude of exposures that result in these adverse outcomes.

**Objective:**

The Study of Asian Women and their Offspring’s Development and Environmental Exposures (SAWASDEE) cohort was established to investigate the impact of prenatal exposure to pesticides on early indicators of cognitive and motor skills, inhibitory control, emotion regulation, and memory that have been found to be important in the development of subsequent neurobehavioral and neurodevelopmental diseases. The overarching goal is to find earlier predictors of potential adverse neurologic outcomes in order to enable earlier interventions that could result in better outcome prognoses.

**Methods:**

Recruitment of this prospective, longitudinal birth cohort began in July 2017 and was completed in June 2019 in Chom Thong and Fang, 2 farming districts in Chiang Mai Province in northern Thailand. Follow-up of the study participants is ongoing. During pregnancy, 7 questionnaires were administered. Time-resolved biospecimen samples were collected monthly (for urine) and during each trimester (for blood) during antenatal care visits. Medical records were abstracted. Infants were administered the NICU Network Neurobehavioral Scale (NNNS) test at 1 month of age. A total of 322 mother-child pairs completed the NNNS test. All children will be followed until 3 years of age and undergo a series of neurodevelopmental tests. We will complete several additional exposure related analyses.

**Results:**

A total of 1298 women were screened, and of those, 394 (30.35%) women were enrolled. The mean gestational age at enrollment was 9.9 weeks (SD 2.6). Differences in literacy were observed between Chom Thong and Fang participants. In Fang, about 54 of 105 (51.4%) participants reported being able to read in Thai compared to about 206 of 217 (94.9%) participants in Chom Thong. The percentages were comparable for reporting to be able to write in Thai.

**Conclusions:**

This longitudinal birth cohort study will inform risk assessment standards for pregnant women in Thailand and other countries. Building awareness of how insecticide exposure during specific windows of pregnancy affects the neurodevelopmental trajectories of children in developing countries is a specific need recognized by the World Health Organization.

**International Registered Report Identifier (IRRID):**

DERR1-10.2196/31696

## Introduction

Prenatal exposure to mixtures of neurotoxic pesticides is prevalent worldwide and has the potential to disrupt and irreversibly alter neurologic development [1-3]. In 2017, approximately 8.8 billion pounds of pesticides were used worldwide [4]. Agricultural workers with occupational exposure to pesticides, on average, have greater exposure than the general population [5]. The agricultural sector comprises approximately 30% of the total workforce in Thailand, and an estimated 70% of the Thai population lives in rural areas [6,7]. Because of their widespread use in Thailand, exposure to pesticides is common [7,8]. The most abundantly used pesticides in Thailand include insecticides, such as pyrethroids and organophosphates (OPs) [8,9], despite their known acute human neurotoxic effects. After exposure to these pesticides via the diet (low background exposure) or dermal absorption and inhalation (primary occupational exposure routes), they are distributed throughout the body or quickly metabolized and excreted [10]. Urinary dialkylphosphates (DAPs), 3,5,6-trichloropyridinol (TCPY), and 3-phenoxybenzoic acid (3PBA) have all been commonly used as biomarkers of OPs and pyrethroid exposure [10].

Previous human and animal studies have reported that following exposure, neurotoxic insecticides, including pyrethroids and OPs, are able to cross the placental barrier and enter the fetal blood stream [11]. Until now, most studies evaluating prenatal pesticide exposure and birth outcomes or neurodevelopment have estimated exposure at only 1 or 2 time points during pregnancy [5]. The literature consistently reports that pesticide exposure is associated with adverse health outcomes; however, the actual outcome measure, exposure measures, and even susceptibility measures vary [5].

The Study of Asian Women and their Offspring’s Development and Environmental Exposures (SAWASDEE) study was conceived to fill some of these knowledge gaps preventing effective policy implementation. The SAWASDEE study is a prospective, longitudinal birth cohort study evaluating pesticide exposure in farmworker women in northern Thailand enrolled during their first trimester of pregnancy and evaluating neurodevelopment in their children at multiple time points until 3 years of age. Our study was designed to collect uniquely refined pesticide exposure data to define critical windows of effect during pregnancy and to evaluate novel neurological testing parameters that could predict a child’s neurodevelopmental trajectory. The study is a multi-institute initiative and builds upon an existing structure of collaboration and capacity building between Emory (Atlanta, GA, USA), Rutgers (Piscataway, NJ, USA), Chiang Mai (Chiang Mai, TH), and Chulalongkorn (Bangkok, TH) Universities. Our cohort includes the collection of robust prenatal and postnatal exposure assessment data; extensive biospecimens, including monthly maternal urine samples and trimester-specific maternal blood; and questionnaire data to enable identification of sensitive windows of exposure to pesticides on development. Children born as part of the birth cohort are followed longitudinally until the age of approximately 3 years to test the hypothesis that prenatal exposure may alter neurodevelopment and that the neurodevelopmental trajectory can be estimated by first-year testing parameters.

Because pesticides are not expected to selectively impact a specific brain region, the study is designed to assess neurodevelopmental trajectories using behavioral measures of infant and early childhood function that reflect underlying neural substrates of visual attention, regulation of emotion, memory, and inhibitory control. This approach integrates the measurement of cognitive and emotional development, recognizing that the regulation of biologically based emotional reactivity, such as anger or fear, is necessary for successful cognitive development. Moreover, the child must develop the ability to delay or inhibit immediate responses in order to achieve higher-level cognitive skills [12].

The purpose of this paper is to provide a comprehensive overview of the SAWASDEE cohort profile as a research resource for potential collaborations, including a description of the data collected, a description of baseline characteristics, and a summary of the future plans for the cohort.

## Methods

### Cohort Development

The SAWASDEE study is based on a longitudinal birth cohort of women residing in northern Thailand who are agricultural workers or live on a working farm. The exposures of interest are pyrethroids and OPs, while the outcomes of interest are birth outcome and child neurodevelopment. All study procedures were reviewed and approved by the institutional review board at Emory University (with Rutgers reliance) and the ethics review committee at the Research Institute for Health Sciences, Chiang Mai University (with Chulalongkorn reliance). Informed consent was obtained from all study participants prior to enrollment.

Recruitment occurred in Chiang Mai Province in northern Thailand because of its robust agricultural sector and its generalizability to other low-/middle-income countries (LMICs) that are similarly reliant on agriculture. The primary crops in Chiang Mai include rice, tangerines, longans, lychee, and ornamental flowers, with their use of pesticides varying greatly with the crop grown. For example, fruits trees require a larger application amount in a shorter application period. Conversely, rice crops require insecticide application more regularly during the growing season but at lower quantities. These 2 types of pesticide application scenarios (ie, high application, short duration, and low application, longer duration) can be easily studied in Chiang Mai Province by focusing on 2 of its distinct districts: (1) Chom Thong and (2) Fang. Chom Thong and Fang are both agricultural communities located about 60 and 150 km, respectively, outside of Chiang Mai City. Chom Thong crops include longans, cut flowers, vegetables, and rice, while in Fang, located in the mountainous region near the Myanmar border, tangerines are the predominant crop. The differences in crops grown in the corresponding districts create a sort of natural experiment of exposure, allowing us to evaluate the differences in these scenarios.

Prior to recruitment, we developed and culturally validated, where appropriate, a detailed set of standard operating procedures (SOPs) and questionnaires. SOPs were developed for sample identification numbers and label generation, biospecimen collection and processing, laboratory testing, and quality assurance and control (QA/QC) procedures, including field blank sample collection, bench-level QC and blank samples, sample compositing, sample randomization for analysis, and neurological testing. In addition, to facilitate sample processing and efficient transfer of samples to Chiang Mai University, we scoped and rented apartments that we outfitted as satellite laboratories we called “SAWASDEE houses” located in each of the districts.

Recruitment began in July 2017 and was completed in June 2019. Women were recruited at community and district hospitals in both districts by trained study nurses. Nurses administered an initial screening questionnaire to determine eligibility of the pregnant women. Women were eligible for participation if they (1) were an agricultural worker or lived within 50 m of an agricultural field, (2) had a Thai identification card permitting hospital and antenatal clinic access, (3) resided in their regional district for ≥6 months and planned residence at least 3 years after delivery, (4) spoke Thai at home, (5) were in good general health (ie, no major medical conditions, such as hypertension, diabetes, thyroid disease, or HIV), (6) consumed fewer than 2 alcoholic beverages per day and did not use illegal drugs [13], (7) and were at <16 weeks’ gestation. Expectant mothers with nonsingleton pregnancies or major pregnancy complications that could affect fetal growth and development were excluded from further participation at the time of diagnosis.

**Figure 1:**
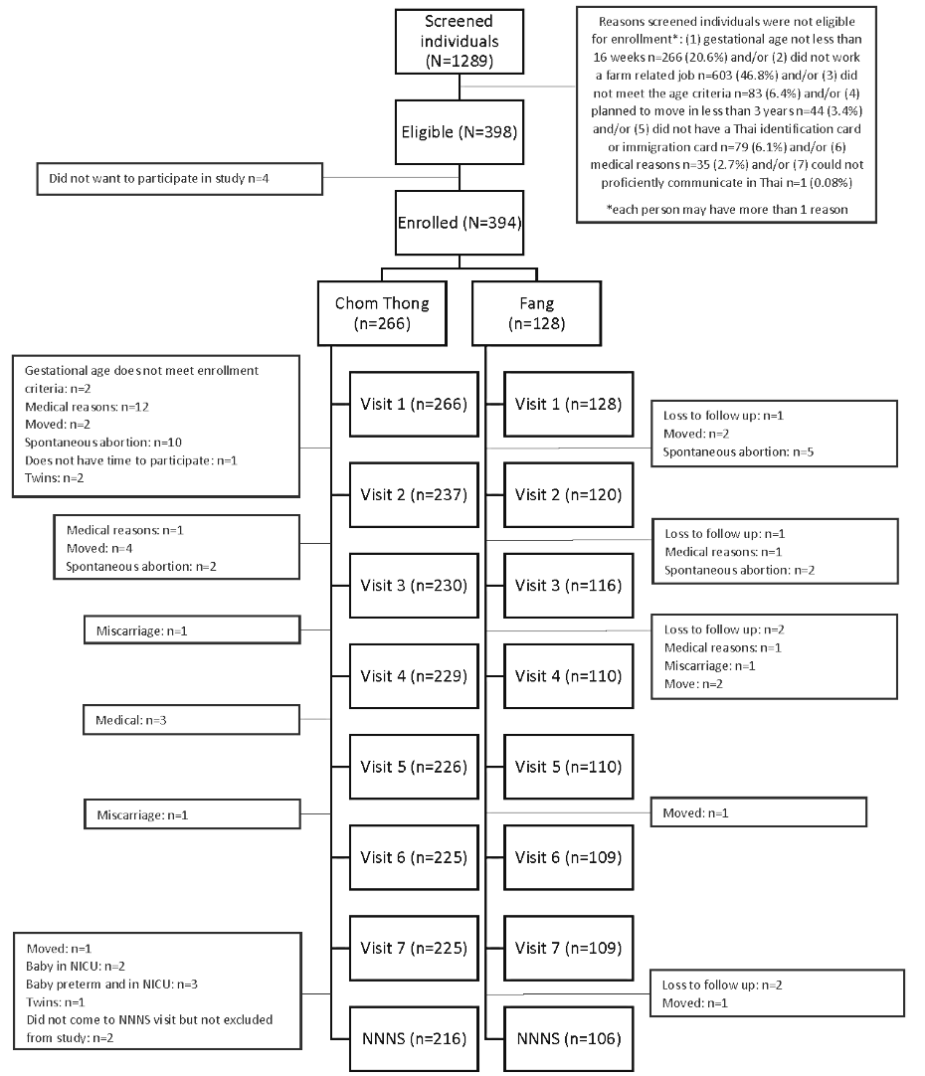
Participant selection and enrollment in the SAWASDEE Birth Cohort Study, Thailand 2017.

We screened 1289 women, and of those, 398 (30.88%) met our enrollment criteria and of which 394 (98.9%) were enrolled. Of these 394 participants, 322 (81.7%) completed all data collection (Figure 1) through their child’s neurological visit at 1 month. Of these 322 participants, 217 (67.4%) were from Chom Thong and 105 (32.6%) were from Fang, with the proportion of total participants reflecting their relative population sizes. Most of the participants who were not retained in the study were excluded due to pregnancy complications (eg, miscarriage, blighted ovum) or because they moved away from the study area.

### Questionnaires

Trained research assistants (RAs) and nurses administered each questionnaire in Thai. These questionnaires included a recruitment survey to determine eligibility (screening questionnaire); an intake questionnaire to collect baseline descriptive statistics; an extensive exposure questionnaire administered during early, middle, and late pregnancy that roughly corresponded to trimesters; and a Knowledge, Attitude, and Practices (KAP) survey of pesticides, collected during the first and third trimesters. The exposure questionnaires, which were validated and used in our pilot study [14], included questions on work-related tasks, household and occupational use of pesticides, personal habits of parents, food consumption, household characteristics and cleanliness, medical histories, maternal nutritional status during pregnancy, and demographics. These questionnaires include detailed information about the population characteristics, including information about medical conditions, medications, weight gain during pregnancy, education, family assets, supplements taken during pregnancy, parity, and more. By administering the exposure questionnaire 3 times, we were able to capture any changes that occurred that might have altered exposures. The KAP survey was used to evaluate the participants’ understanding of the toxicity and potential effects of pesticides and their routine practice related to pesticides and how those practices changed as pregnancy progressed. After delivery, an additional 9 questionnaires were completed over the course of 36 months to ascertain developmental milestones, maternal intelligence quotient (IQ), family assets and the home environment, and anthropometrics of the children to evaluate their nutritional status.

### Case Records

The following information was extracted from medical records: early pregnancy weight and weight gain, parity, past obstetric history, planned pregnancy, smoking and alcohol consumption, marital status, occupation, history of illness, and prenatal visit history, which included gestational age at first visit, subsequent visit dates, doctor-assessed nutritional status, and compliance with micronutrient supplementation regimens. These data were updated at each of the visits to track a variety of indicators, including maternal nutritional status (using weight gain as a surrogate) and significant stressors that could complicate the exposure-effect evaluation. Data extracted from participant birth records included sex, birth weight, length, gestational age, and head circumference; appearance, pulse, grimace, activity, and respiration (APGAR) scores; type and route of delivery; anesthesia; and information about maternal and neonatal complications.

### Biospecimen Collection

Biospecimens collected from enrolled participants are shown in Table 1. Most of these samples were processed and archived for planned future analysis or novel research questions.

Blood, serum, urine, and hair were used for exposure assessment, with urine being the primary matrix used. Blood was used for susceptibility biomarker analysis (ie, paraoxonase 1 [PON1] phenotyping), and placenta tissue was used for transcriptomics analysis.

**Table 1:** Questionnaire, biological sample collection, and neurotesting, SAWASDEE^a^ study, Thailand, 2017-2019.

Time point	Visit, n	Questionnaire	Biological sample collection (M=maternal, C=child)									Neurological testing^b^
			Urine	Swabs^c^	Blood	Cord blood	Meconium	Breast milk^d^	Placenta	Hair^e^		
Enrollment <12 weeks	1	Intake, KAP^f^	M	M	M	N/A^g^	N/A	N/A	N/A	M	N/A	
16 weeks	2	Maternal exposure (ME) baseline	M	N/A	N/A	N/A	N/A	N/A	N/A	N/A	N/A	
20 or 22 weeks	3	N/A	M	M	M	N/A	N/A	N/A	N/A	N/A	N/A	
28 weeks	4	ME 2nd trimester	M	N/A	N/A	N/A	N/A	N/A	N/A	N/A	N/A	
32 weeks	5	KAP	M	M	M	N/A	N/A	N/A	N/A	N/A	N/A	
36 weeks	6	ME 3rd trimester	M	N/A	N/A	N/A	N/A	N/A	N/A	N/A	N/A	
Delivery	7	Medical chart abstraction	N/A	N/A	N/A	M	N/A	N/A	M	N/A	N/A	
1-3 days postdelivery	8	DASS^h^	C	N/A	N/A	N/A	C	N/A	N/A	N/A	NNNS^i^	
4 months	10	DASS	C	N/A	N/A	N/A	N/A	M	N/A	N/A	Visual habituation	
7 months	11	DASS	C	N/A	N/A	N/A	N/A	M	N/A	N/A	Visual habituation, Bayley’s Scales of Infant Development (BSID) motor, visual paired comparison (VPC), visual expectation (VEP), continuous familiarization (CFT), A not B test (AB)	
12 months	12	Child exposure (CE)	C	N/A	N/A	N/A	N/A	M	N/A	N/A	VPC, VEP, CFT, AB, delayed recall, deferred imitation, cross-modal, Labtab subtests	
12 months	12	Infant Behavior Questionnaire (IBQ), DASS	N/A	N/A	N/A	N/A	N/A	N/A	N/A	N/A	N/A	
12 months	12	HOME^j^	N/A	N/A	N/A	N/A	N/A	N/A	N/A	N/A	N/A	
18 months	13	BDI, TONI^k^-IV	C	N/A	N/A	N/A	N/A	N/A	N/A	N/A	VPC, VEP, CFT, AB, delayed recall, deferred imitation, cross-modal	
24 months	14	DASS, CE, HOME, Language Development Survey	C	N/A	N/A	N/A	N/A	N/A	N/A	N/A	Delayed recognition, deferred imitation, cross-modal, inhibitory control	
36 months	15	DASS, Child Behavior Checklist (CBCL), Early Child Behavior Questionnaire (ECBQ), CE	C	N/A	N/A	N/A	N/A	N/A	N/A	N/A	Bayley III	

^a^SAWASDEE: Study of Asian Women and their Offspring’s Development and Environmental Exposures.

^b^A neurological test for this time point is scheduled for 2 sessions, 2 weeks apart, but only 1 sample will be collected from either session.

^c^Swabs: oral, vaginal, and gut.

^d^If breastfeeding at visit time.

^e^Need 5 g of hair.

^f^KAP: Knowledge, Attitude, and Practices.

^g^N/A: not applicable.

^h^DASS: Depression Anxiety Stress Scale.

^i^NNNS: NICU Network Neurobehavioral Scale.

^j^HOME: Home Observation for the Measurement of the Environment.

^k^TONI: Test of Nonverbal Intelligence.

### Exposure Assessment

To provide trimester-specific exposure measures during pregnancy, while still keeping the study costs reasonable, we created trimester-specific urine composites from each participant but still retained the original discrete samples for later use. DAPs, TCPY, and 3PBA were measured in these maternal composited urine samples (Table 2) using established analytical methods [15-17]. PON1 enzyme activity, a measure of OP insecticide susceptibility, was measured in maternal serum using previously established procedures [14]. Other samples were archived for future use.

**Table 2:** Measurements to be made in biological matrices or matrices to be archived for future analysis, SAWASDEE^a^ study, Thailand, 2017-2019.

			Analytes		Matrix		Laboratory
**Insecticide or measure**							
	OP^b^	DAP^c^ metabolites		Maternal urine		Chiang Mai University (CMU)	
	OP	Chlorpyrifos (TCPY^d^)		Maternal urine		Emory	
	Pyrethroid	3PBA^e^		Maternal urine		Emory	
	OP	PON1^f^ enzyme activity		Maternal blood		CMU	
	mRNA	N/A^g^		Placenta		Mount Sinai	
**Preserved samples**							
	Organochlorines/metals	Dichlorodiphenyldichloroethylene/dichloro-diphenyl-trichloroethane (DDE/DDT), lead, mercury		Maternal blood		Emory	
	OP/pyrethroids	DAPs/3PBA		Child urine		CMU/Emory	
	OP/pyrethroids	Chlorpyrifos, permethrin, cypermethrin		Meconium, breast milk, blood/plasma		CMU/Emory	
	DNA methylation	N/A		Preserved DNA		To be determined (TBD)	
	Microbiome (maternal vaginal and gut and child gut)	N/A		Vaginal/rectal swabs		TBD	

^a^SAWASDEE: Study of Asian Women and their Offspring’s Development and Environmental Exposures.

^b^OP: organophosphate.

^c^DAP: dialkylphosphate.

^d^TCPY: 3,5,6-trichloropyridinol.

^e^3PBA: 3-phenoxybenzoic acid.

^f^PON1: paraoxonase 1.

^g^N/A: not applicable.

### Assessment of Neurological Outcomes

Extensive neurodevelopment examination of each child was conducted by trained neuropsychologists at the following time points: 1, 4, 7, 12, 18, 24, and 36 months (Table 3).

Assessment at these intervals represents a significant departure from previous studies of pesticide exposure and will allow examination of how pesticides may alter the neurodevelopmental trajectory of children. For tests administered at more than 1 developmental period, age-appropriate adjustments were made to increase the difficulty of the test. Infant and child responses were either scored in real time in the case of visual habituation tests or scored from digital recordings when testing was complete. Interrater reliabilities were conducted monthly for all tests using different videos each month and independent scoring by psychologists to maintain reliability among testers. All tests were routinely recorded to allow recheck procedures and scoring. The neurodevelopmental measures used in our study included those traditionally used in other birth cohort studies (eg, Bayley Scales of Infant and Toddler Development at age 3 years) but also included tests of visual habituation at 4 and 7 months as indicators of visual attention and information processing, followed by tests of infant memory and executive function (Tables 1 and 3). The NNNS was used within the first week of delivery and is a comprehensive assessment of both neurological integrity and behavioral function. Additionally, tests of emotion regulation and inhibitory control were administered to determine how not only cognitive skills but also emotional development may be altered by exposures to pesticides. This more comprehensive battery of tests will enhance our ability to determine whether and at what developmental period prenatal pesticide exposure alters the building blocks of later cognitive, motor, and emotional development.

**Table 3:** Description of neurological test, the domain of function assessed by the test, and age of administration, SAWASDEE^a^ study, Thailand, 2017-2019.

Domain/test			Age (months)		Task description		Measure
**Newborn neurological state regulation**							
	Behavioral Assessment Scale	Newborn		Interactive assessment of physiologic and motor organization arousal and ability with stimulation; ability to attend and remain alert		Habituation, orientation/attention, motor control, range of state, regulation of state, autonomic stability, reflexes	
	NNNS^b^	1		N/A^c^		N/A	
**Visual attention**							
	Visual habituation	4, 7, 10		Familiarization with a visual stimulus followed by pairing with a novel stimulus		Visual habituation, duration of first look, novelty preference	
	Visual paired comparison (VPC)	18, 30		5 faces and 4 abstract figure patterns during familiarization with 2 identical images; tested by pairing with a novel stimulus		Look duration, shift rate, novelty preference	
**Processing speed**							
	Visual expectation paradigm	18, 30		Orientation to target stimuli presented to L and R of midline with 10 baseline and 60 predictable trials		Mean reaction time	
**Encoding speed**							
	Continuous familiarization (CFT) task	18, 30		Paired faces presented, 1 of which changes from trial to trial while the other remains constant		Trials to criterion (consistent preference for novel target), look duration, shift rate	
**Memory**							
	Immediate recognition	18, 30		Derived from VPC		Mean novelty preference score (percentage time looking at novel stimulus), look duration, shift rate	
	Delayed recognition	18, 30		Familiarization with 3D object followed by visual identification when paired with new object		Mean novelty preference score	
	Deferred imitation	10, 18, 24, 30		Modeling series of events with objects with immediate and delayed reproduction of sequence		Mean percentage of target actions reproduced in correct order	
**Representational competence**							
	Tactual-visual cross-modal transfer	18, 30		Tactile familiarization with 3D object followed by visual identification when paired with new object		Mean novelty preference score, look duration, shift rate	
**Executive function**							
	A not B test (AB), looking version	10, 24		Toy hidden in counterbalanced hiding locations based on performance		Percentage correct trials	
**Language and overall development**							
	Bayley Scales of Infant and Toddler Development-III	36		Sensorimotor development, exploration and manipulation, concept formation, memory, receptive/expressive communication, perceptual motor integration, motor speed, planning, locomotion, coordination		Cognitive scaled score, gross motor scaled score, fine motor scaled score, receptive language scaled score, expressive language scaled score	
	Language Development Survey	24		Parent report of child's individual word and phrase expressions		Number of words, length of phrases	
	HOME^d^	12, 24		Structured, observational interview of parental interaction with child and overall environment		Responsivity, acceptance, organization, learning materials, involvement, variety	
**Emotion regulation and adjustment**							
	Labtab subtests: 1. Fear (a) spider, (b) masks; 2. Anger (c) maternal separation, (d) toy behind barrier; 3. Joy (e) puppets	12		Remote-controlled spider approaches child; masks displayed 1 at a time; mom leaves room for 30 seconds; toy that child has been playing with placed behind barrier; standardized dialogue between puppets presented by examiner		Occurrence and intensity of behaviors coded from tape (latency to response, eg, fear); intensity of physical and verbal responses (eg, facial, vocal, body)	
	Early Childhood Behavior Questionnaire (ECBQ)	12		Parent report checklist to survey child behavior		Parent rating of child behavior: negative affect, surgency-exhaust, effort control	
	Child Behavior Checklist (CBCL)	36		Primary caregiver report of behaviors during past 2 months		Emotionally reactive, anxious/depressed, somatic complaints, withdrawn, attention problems, aggressive behavior, sleep problems	
**Inhibitory control**							
	Snack delay	24		Child waits until instructed to retrieve a snack		Ability to delay in seconds	
	Crayon delay	24		Child gives crayons to draw but must wait		Latency to touch crayons	
	Toy prohibition by mother	24		Child in room full of toys but told by mother not to play with the toys		Frequency and type of mother intervention, frequency of child attention to toy (eg, look, touch)	

^a^SAWASDEE: Study of Asian Women and their Offspring’s Development and Environmental Exposures.

^b^NNNS: NICU Network Neurobehavioral Scale.

^c^N/A: not applicable.

^d^HOME: Home Observation for the Measurement of the Environment.

The Home Observation for the Measurement of the Environment (HOME) is a structured interview conducted with the primary caretaker and child in the home of each participant to observe and assess the physical environment, family structure/relationships, and learning environment for the child [18]. HOME was administered at 12 and 24 months. HOME has been extensively used across the world, with adaptations for different cultures, and is predictive of child cognitive development [19]. Mothers were administered the Test of Nonverbal Intelligence (TONI)-I as a nonverbal measure of intelligence in recognition of the effects the mother’s education and cognitive abilities have on child neurodevelopment. The Depression Anxiety Stress Scale 21 (DASS-21) is a measure of maternal depression and anxiety that has been used in cross-cultural settings, including Asia [20]. The DASS-21 was administered at the first antenatal visit (1 week after birth) and at every subsequent child assessment visit to assess the known effects of maternal depression and anxiety on development. Although we asked families to estimate their monthly income, in low- and middle-income agricultural settings, monthly income estimates are difficult to determine and may not fully capture the economic health of a family. Therefore, based on the Economic and Social Research Council (ESRC) Research Group on Wellbeing in Developing Countries [21], we developed a family assets questionnaire to augment income estimates [22].

### Statistical Analysis

#### Calculation of Power

We assumed that differences in neurobehavioral estimates for infants of mothers working on fruit/vegetable farms and those with mothers working on rice farms will be similar to differences observed between infants from preterm versus full-term pregnancies. Thus, Rose et al [23] provided preliminary estimates, from measurements observed at 24 months, on which to determine sample sizes. Table 4 provides the outcomes and estimated total sample sizes (for 1 sample) needed to find a difference between groups with 80% power using a 2-sided 2-group *t* test at the .05 significance level. The longitudinal design of our study provides further power than estimated. Based on these calculations, 300 infants should provide enough power to detect differences between groups.

**Table 4:** Total sample sizes needed to detect differences in outcomes with 80% power.

Neurodevelopmental outcomes			Full-term/lower exposure, mean (SD)		Preterm/higher exposure, mean (SD)		n
**Memory**							
	Immediate recognition Rose visual paired comparison (VPC) task percentage novelty	57.05 (5.38)		55.33 (4.36)		258	
	Immediate recognition Fagan VPC task percentage novelty	59.55 (4.82)		57.22 (3.65)		108	
	Recall: elicited imitation (percentage correct)	59.3 (18.65)		52.08 (21.19)		242	
**Processing speed**							
	Encoding speed (trails to criterion)	8.7 (4.12)		11.47 (8.5)		186	
**Attention**							
	Mean look duration (composite)	–0.1 (0.58)		0.19 (0.79)		182	
	Shift rate (composite)	0.07 (0.72)		–0.15 (0.7)		330	

#### Cohort Characteristics

The baseline characteristics of the study participants were analyzed and reported by study location (Chom Thong and Fang). All analyses were performed using SAS V.9.4 (SAS Institute Inc., Cary, NC, USA).

## Results

### Participant Characteristics

In total, 1289 pregnant women were administered the recruitment-screening questionnaire. The primary reasons screened individuals were not eligible were as follows: (1) their gestational age was more than 16 weeks (n=266, 20.64%) or (2) they were agricultural workers or lived within 50 m of an agricultural field (n=603, 46.78%). Of those individuals eligible for the study (n=398, 30.88%), 394 (98.9%) women provided consent to join the study and were enrolled (Figure 1). Among the participants, 322 (81.7%) completed the study through the first neurodevelopmental examination of their child, the NNNS.

Of the 322 (Chom Thong n=216, 67.1%; Fang n=106, 32.9%) mother-child pairs with a completed NNNS, the mean age of SAWASDEE study participants at enrollment was 25 years (SD 5.3); see Table 5. The mean gestational age at enrollment was 9.9 weeks (SD 2.6 weeks). In Chom Thong, 47 of 216 (21.8%) and 165 of 216 (76.4%) women reported being legally married or living as married, respectively, compared to 1 of 106 (0.9%) and 102 of 106 (96.2%) women in Fang. The mean gestational age at birth was 38.5 weeks (SD 1.2 weeks), with gestational age determined by ultrasound or last menstrual period differing by participant site (n=105/216, 48.6%, and n=106/106, 100%, determined by ultrasound at Chom Thong and Fang, respectively). The mean infant birth weight was 3 kg (range 1.7-4.2 kg) and was not statistically significant (*P*<.05) between participants from Chom Thong and Fang.

The median and range of exposure measures and PON1 activity categorizations are shown in Table 6. Overall, exposure to OPs was higher in Fang than in Chom Thong in keeping with their crop application rates. Levels of pyrethroids were similar across sites. Overall, about 29 (9%), 126 (39.1%), and 167 (51.8%) of 322 participants had low, normal, and high PON1 activity, with more participants in Fang having normal activity than in Chom Thong.

**Table 5:** Descriptive characteristics of the SAWASDEE^a^ cohort, Thailand, 2017-2019.

Variable		SAWASDEE (N=322)	Chom Thong (n=216)	Fang (n=106)
Age of mother at enrollment (years), mean (SD)		25.0 (5.3)	25.5 (5.3)	23.9 (5.0)
Body mass index (BMI) of mother at visit 1 (kg/m^2^), mean (SD)		22.9 (5.3)	22.7 (4.9)	23.3 (6.2)
Missing, n (%)		1 (0.03)	0	1 (0.9)
**Ethnicity of the mother, n (%)**				
	Thai	204 (63.4)	196 (90.7)	8 (7.5)
	Hmong	10 (3.1)	10 (4.6)	0
	Thai Yai	25 (7.8)	1 (0.5)	24 (22.6)
	Karen (Pagayo)	6 (1.9)	6 (2.8)	0
	Burmese	4 (1.2)	2 (0.9)	2 (1.9)
	Akha	5 (1.5)	0 (0.0)	5 (4.7)
	Pa-Long (Dara-ang)	36 (11.2)	0 (0.0)	36 (34)
	Lahu	31 (9.6)	0 (0.0)	31 (29.2)
	Lawa	1 (0.3)	1 (0.5)	0
**Mother, highest education level completed, n (%)**				
	None, never attended school	58 (18.4)	12 (5.6)	46 (43.1)
	Primary 1-6	47 (14.4)	19 (8.9)	28 (26.1)
	Junior high/high school	106 (32.4)	86 (39.6)	20 (18.3)
	High school/did not graduate	70 (21.4)	63 (29.3)	7 (6.2)
	Diploma/technical school equivalent	28 (8.4)	24 (11.4)	4 (3.8)
	Attended college but did not graduate	1 (0.4)	1 (0.6)	1 (0.9)
	College graduate or more	12 (3.4)	11 (5.5)	0
**Mother able to read Thai, n (%)**				
	Yes	260 (80.4)	206 (95.1)	54 (50.9)
	No	62 (19.4)	10 (4.9)	52 (49.1)
**Mother able to write in Thai, n (%)**				
	Yes	262 (81.4)	205 (94.3)	57 (53.8)
	No	60 (18.4)	11 (5.7)	49 (46.1)
**Household income (Thai baht)^b^, mean (SD)**		10615.1 (8336.9)	11572.4 (9499.0)	8709.6 (4818.4)
	Missing, n	11	9	2
**Number of people living in households, mean (SD)**		5.2 (2.5)	4.9 (2.2)	5.7 (3.0)
	Missing, n	22	15	7
**Marital status, n (%)**				
	Legally married	47 (14.4)	46 (21.9)	1 (0.9)
	Living as married	268 (83.4)	166 (76.6)	102 (96.2)
	Widowed	2 (0.4)	2 (0.2)	0
	Divorced	2 (0.4)	2 (0.2)	0
	Separated	3 (0.4)	0	3 (2.8)
**Mother working in agriculture during pregnancy, n (%)^c^**				
	Yes	258 (80.4)	182 (84.3)	76 (72.4)
	No	63 (19.6)	34 (15.7)	29 (27.6)
	Missing, n	1	0	1
**Maternal smoking during pregnancy, n (%)**				
	Yes	4 (1.3)	2 (0.9)	2 (1.9)
	No	317 (98.7)	214 (99.1)	103 (98.1)
	Missing, n	1	0	1
**Gestational age (weeks), mean (SD)**				
	At enrollment	9.9 (2.6)	9.7 (2.6)	10.2 (2.5)
	Missing, n	3	0	3
	At birth	38.5 (1.2)	38.5 (1.1)	38.4 (1.2)
	Missing, n	5	4	1
**Infant birth weight (kg), mean (SD)**		3.0 (0.4)	3.0 (0.4)	2.9 (0.4)
	Missing, n	2	2	0
**Infant's sex, n (%)**				
	Male	159 (49.5)	105 (48.8)	54 (50.4)
	Female	162 (50.5)	110 (51.2)	52 (49.5)
	Missing, n	1	1	0

^a^SAWASDEE: Study of Asian Women and their Offspring’s Development and Environmental Exposures.

^b^A currency exchange rate of 1 Thai baht = US $0.03 is applicable.

^c^Ever/never variable, participants asked in each trimester, and if they ever responded yes, they were considered yes.

**Table 6:** Concentrations of exposure biomarkers in the SAWASDEE^a^ study, 2017-2022 (N=322).

Biomarker			SAWASDEE (N=322)			Chom Thong (n=216)			Fang (n=106)	
		Frequency of detection (FOD), %		Median (range)	FOD, %		Median (range)	FOD, %		Median (range)
**Exposure measures**										
	*Σ*DAP^b^ (nmol/L)	100		86.6 (42.6-2852)	100		79.7 (42.6-2852)	100		150 (43.9-1942)
	*Σ*DEAP^c^ (nmol/L)	100		46.9 (7.3-1714)	100		40.4 (7.3-511)	100		98.3 (8.6-1714)
	*Σ*DMAP^d^ (nmol/L)	48		35.5 (35.31-2758)	67		35.5 (<35.3-2758)	47		<35.3 (<35.3-275)
	TCPY^e^ (ng/mL)	98		5.2 (<0.31-41.3)	100		5.1 (0.61-41.3)	94		5.36 (<0.31-40.1)
	3PBA^f^ (ng/mL)	84		0.58 (<0.31-19.8)	86		0.61 (<0.31-6.1)	80		0.51 (<0.31-19.8)
**PON1^g^ activity**										
	Low	9		N/A^h^	9		N/A	8		N/A
	Normal	39		N/A	37		N/A	45		N/A
	High	52		N/A	54		N/A	47		N/A

^a^SAWASDEE: Study of Asian Women and their Offspring’s Development and Environmental Exposures.

^b^DAP: dialkylphosphate (*Σ*DAP=sum of all DAP metabolites).

^c^DEAP: diethyl alkylphosphate (*Σ*DEAP=sum of all DEAP metabolites).

^d^DMAP: dimethyl alkylphosphate (*Σ*DMAP=sum of all DMAPs).

^e^TCPY: 3,5,6-trichloropyridinol.

^f^3PBA: 3-phenoxybenzoic acid.

^g^PON1: paraoxonase 1.

^h^N/A: not applicable.

## Discussion

### Principal Findings

The SAWASDEE study was successfully established to fill critical gaps in the literature by measuring exposure multiple times during each trimester of pregnancy, while tracking neurodevelopmental trajectories in infancy and early childhood. By establishing the association between exposure biomarkers during early-to-late pregnancy and these neurodevelopmental trajectories, we will be able to examine windows of increased susceptibility to pesticide exposure. This information may result in specification of vulnerability, resulting in recommendations for reduction in adverse neurodevelopmental outcomes.

Our enrollment and participant rates were notable, provided the low population density and complexities of travel in these regions. The terrain is mountainous and winding; participants had to often rely on public transportation to make the study visits. We tried to align our study visits as closely as possible to their antenatal visits for convenience and reimbursed participants for travel. However, in some of the worst cases, participants traveled for up to 4 hours to attend these visits. Their loyalty to our study was largely a result of the close relationship our study nurses and RAs developed with the participants and the immense trust our participants had in them. Although we believe that the relationships that were established between field staff and study participants were essential in preserving study retention, it is also possible there were behavioral changes in pesticide practices over time due to unintentional influence by field staff or involvement in the study. Analysis of the KAP questionnaires from our pilot study revealed that there was an increase in pesticide-related knowledge among pregnant women and that influenced changes in pesticide practices [24]. In this study, we did observe that many women stop working in agricultural-related fields late in pregnancy; however, we were unable to discern whether this is a result of the physical challenges associated with pregnancy, especially in the third trimester of pregnancy; a natural shift in agricultural tasks required; or behavioral change from participating in our study. We hope to disentangle the reason(s) for a reduction in agricultural work late in pregnancy in future analyses using the longitudinal KAP data collected in our study.

### Limitations

Our study is not without limitations. We may have some differential bias in gestational age among sites, which we described above. There is potential for exposure misclassification because of the contribution of preformed environmental metabolites to the measured urinary metabolite levels. However, given that our population is experiencing occupational exposures that occur after known pesticide application events, we expect this contribution to be smaller than if we were evaluating background dietary exposures. We also did not measure all pesticides to which participants may have been exposed. Although we have basic dietary information for mothers during pregnancy, we could not conduct a thorough assessment of dietary information, such as a food frequency questionnaire, largely because of funding issues. In addition, some of our neurological tests have no known cultural norms for comparison, but our pilot studies have provided insight into how this can be addressed in our data analyses [14].

Despite our study’s limitations, this is still the first study to evaluate these neurodevelopmental outcomes in Thailand and to provide refined exposure measurements during pregnancy.

### Strengths

Our study has several strengths. We have time-resolved and highly sensitive exposure measures from all trimesters of pregnancy in our participants. This includes 2 distinct exposure scenarios: long-term, lower-dose pesticide exposure, and high-dose, intermittent pesticide exposure. The robustness of exposure and outcome data will allow investigators to evaluate numerous scientific questions and ultimately improve the generalizability of associations between pesticide exposure and neurodevelopmental outcomes. The majority of previous studies have been conducted using cohorts from high-income countries and examination in LMICs is lacking. The success of our study recruitment and retention is largely attributed to the strong community-centered nature and the respect shown to the field research staff by study participants. We found this to be especially true within the Thai culture—individuals have a high level of respect for health care practitioners. The study also includes highly refined neurodevelopment assessment with sensitive measures of early neurointegrity that measure the basis of later cognitive and motor development. Previous studies have used clinical measures that are relatively less sensitive to subtle and early adverse effects. We can evaluate windows of vulnerability as they relate to specific neurodevelopmental processes and the trajectory of development. In addition, this study assesses not only cognitive and motor skills but also inhibitory control and emotion regulation, not previously studied with behavioral measures. The NNNS, in addition to the other neurodevelopmental assessments included in the study, creates a robust database of neurodevelopment, which can be used to evaluate several scientific questions related to neurodevelopment, including evaluating trajectories of neurodevelopment. The prospective longitudinal data in our study present a valuable resource for evaluating many scientific questions. The SAWASDEE study offers potential collaborators an opportunity to work with robust estimates of exposure and neurodevelopment. The number of supplemental studies that are part of the SAWASDEE study continues to increase, and thus, the database continues to grow, offering investigators an opportunity to test several hypotheses.

### Conclusion

The SAWASDEE birth cohort study provides information essential for risk assessment paradigms addressing the risk of prenatal pesticide exposure and neurodevelopment. The cohort is unique in that it is designed with highly refined exposure and neurodevelopmental data with the ability to determine neurodevelopmental trajectories early in infancy with the hope of early intervention. Successful enrollment followed by high rates of retention and participation in the study provides a unique opportunity for evaluating maternal pesticide exposure and child neurodevelopmental outcomes.

## Abbreviations

3PBA: 3-phenoxybenzoic acid
DAP: dialkylphosphate
HOME: Home Observation for the Measurement of the Environment
IQ: intelligence quotient
KAP: Knowledge, Attitude, and Practices
LMIC: low-/middle-income country
NNNS: NICU Network Neurobehavioral Scale
OP: organophosphate
PON1: paraoxonase 1
QA/QC: quality assurance and control
RA: research assistant
SAWASDEE: Study of Asian Women and their Offspring’s Development and Environmental Exposures
SOP: standard operating procedures
TCPY: 3,5,6-trichloropyridinol
